# Supplementary figures and images for: OPA1 supports mitochondrial dynamics and immune evasion to CD8+ T cell in lung adenocarcinoma
Source: PeerJ. 2022 Dec 21;10:e14543. doi: 10.7717/peerj.14543 (PMC9789695; doi:10.7717/peerj.14543)

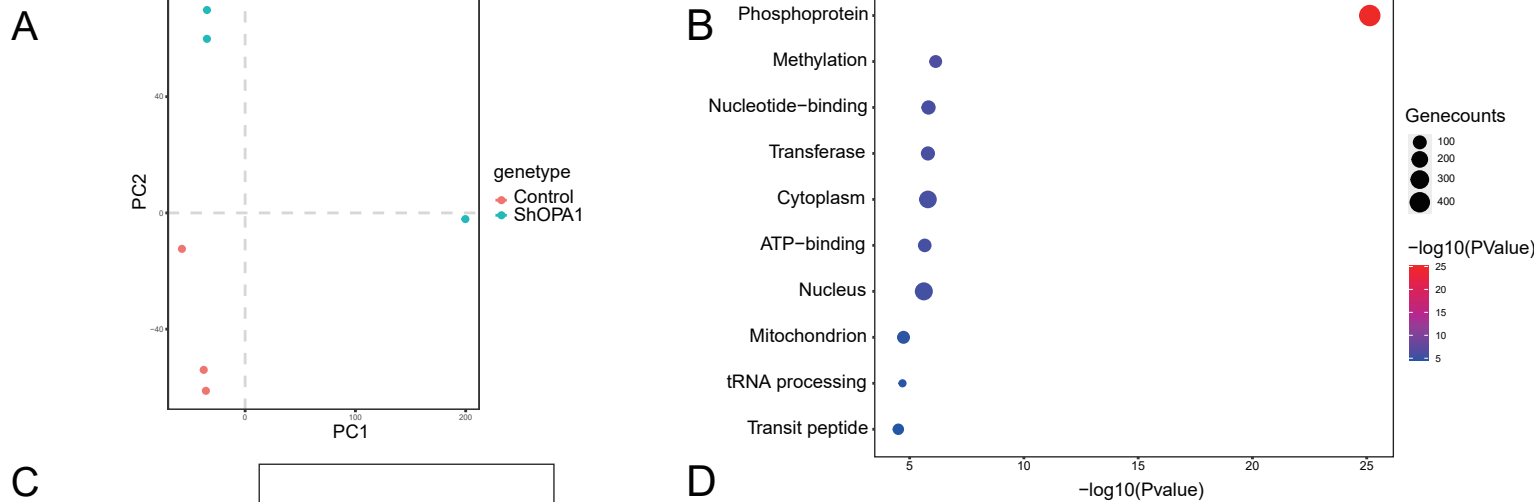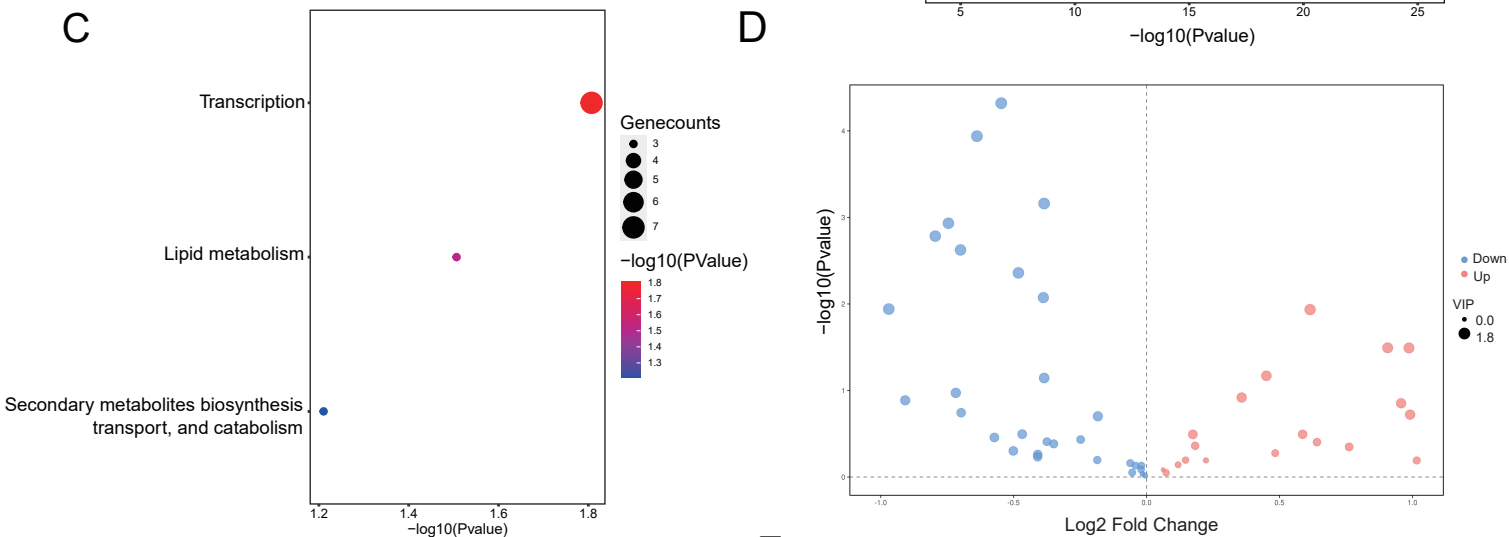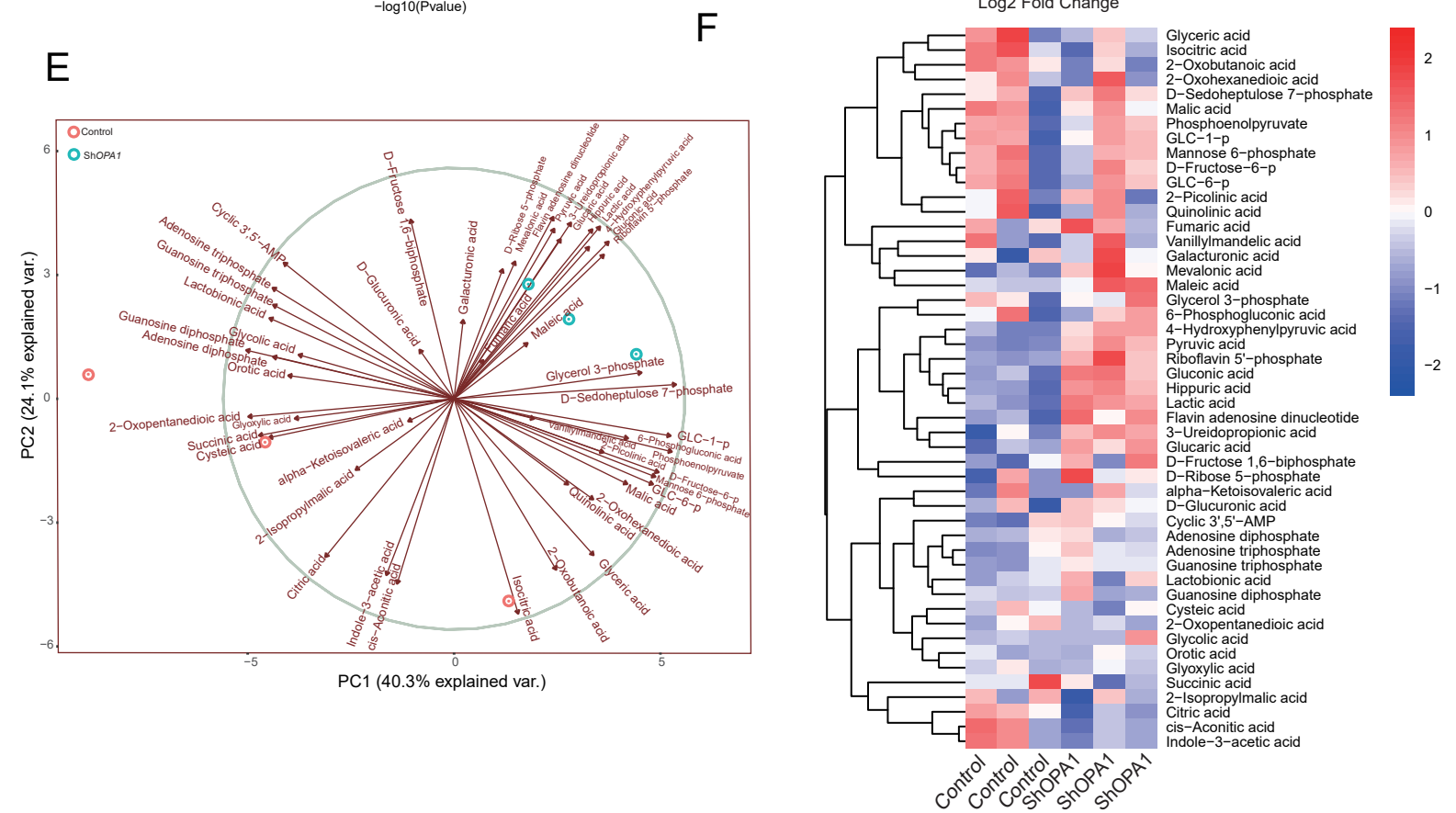

Supplement: Supplemental Information 2 — (A) RNA-seq data were analyzed by PCA. (B) KEGG pathway enrichment analysis within the complete set of differentially expressed genes. (C) COG_ONTOLOGY pathway enrichment analysis within the complete set of differentially expressed genes. (D) Volcano plot of the metabolites between Control and ShOPA1 cells. (E) Data of the differential metabolites were analyzed by PCA, and different colors of the points represent different sample grouping information, and the arrow direction represents the sample content information of the corresponding substance in the surrounding area. (F) Heatmap for unsupervised clustering of the all the metabolites involved. [file peerj-10-14543-s002.pdf]

**A**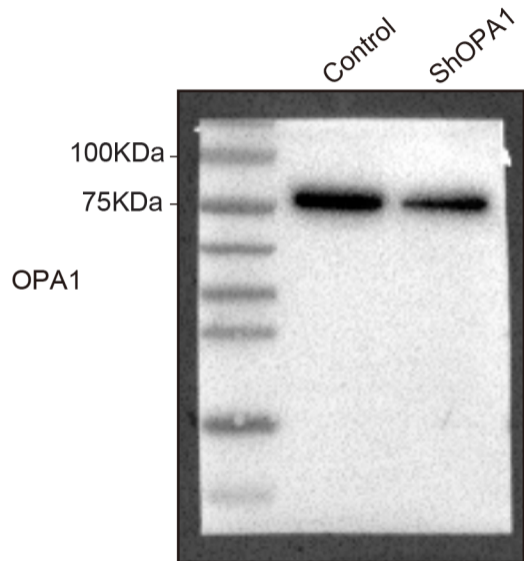**B**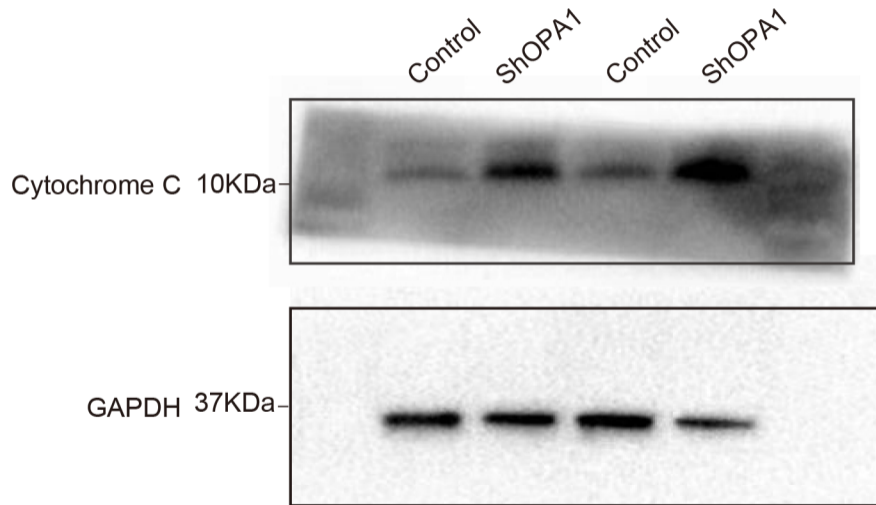

Supplement: Supplemental Information 3 [file peerj-10-14543-s003.pdf]

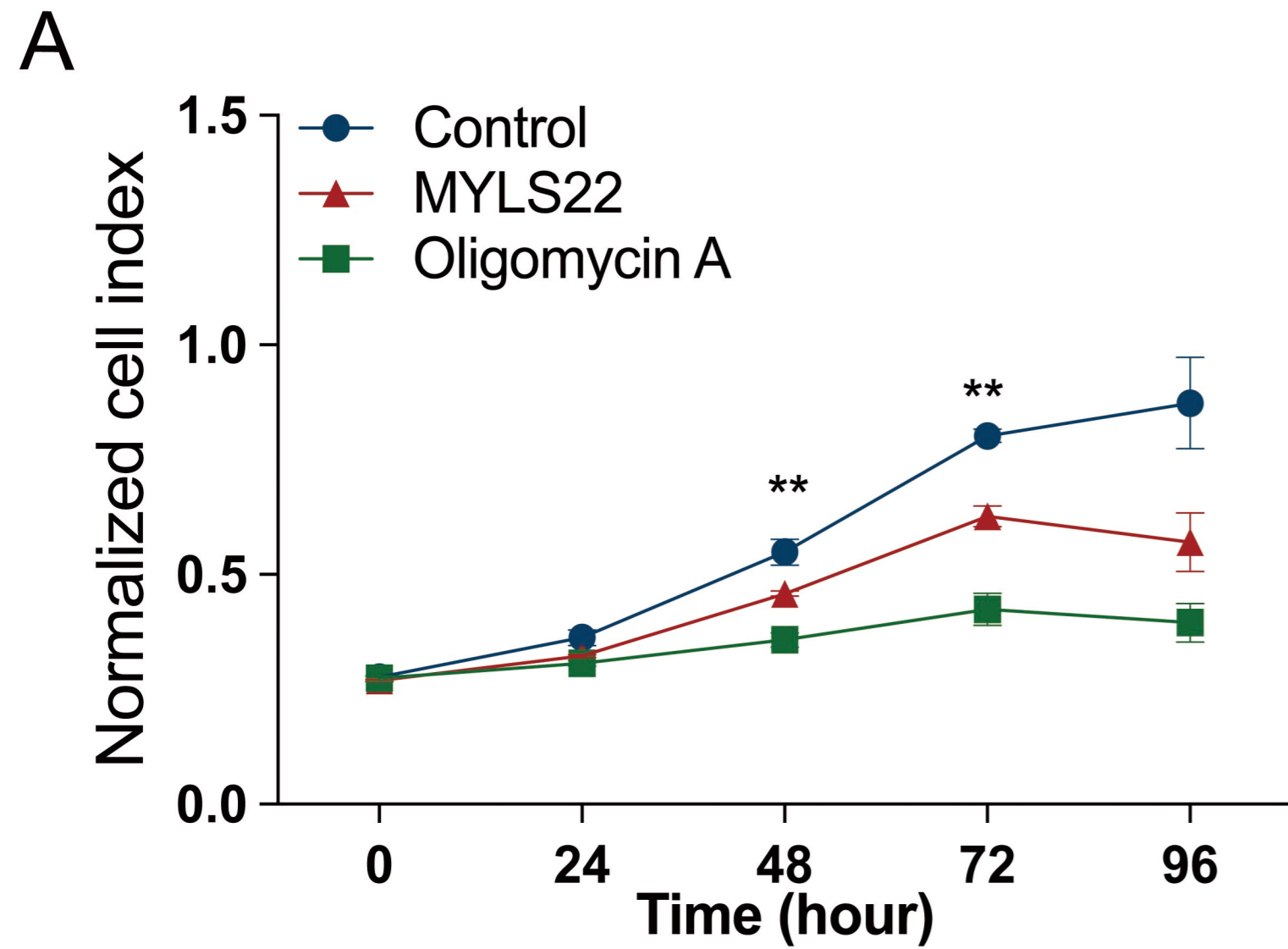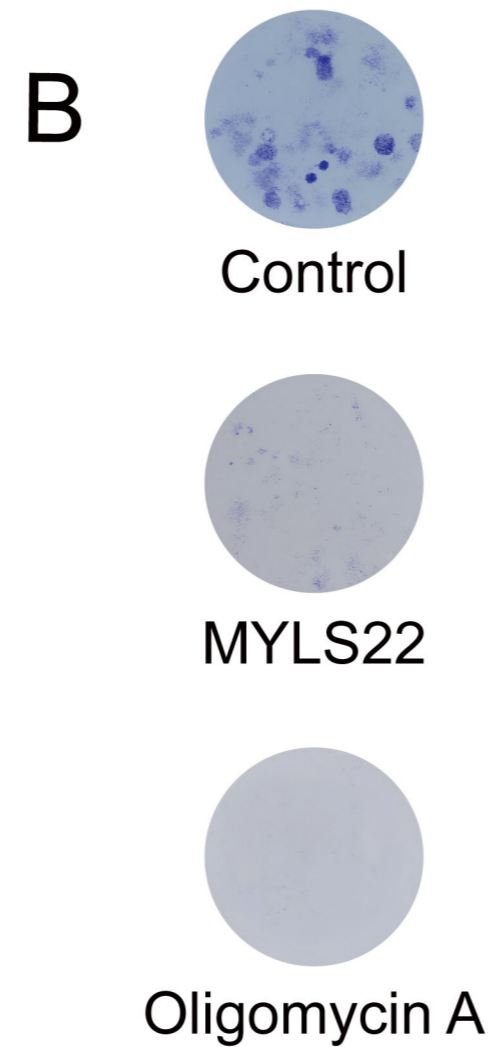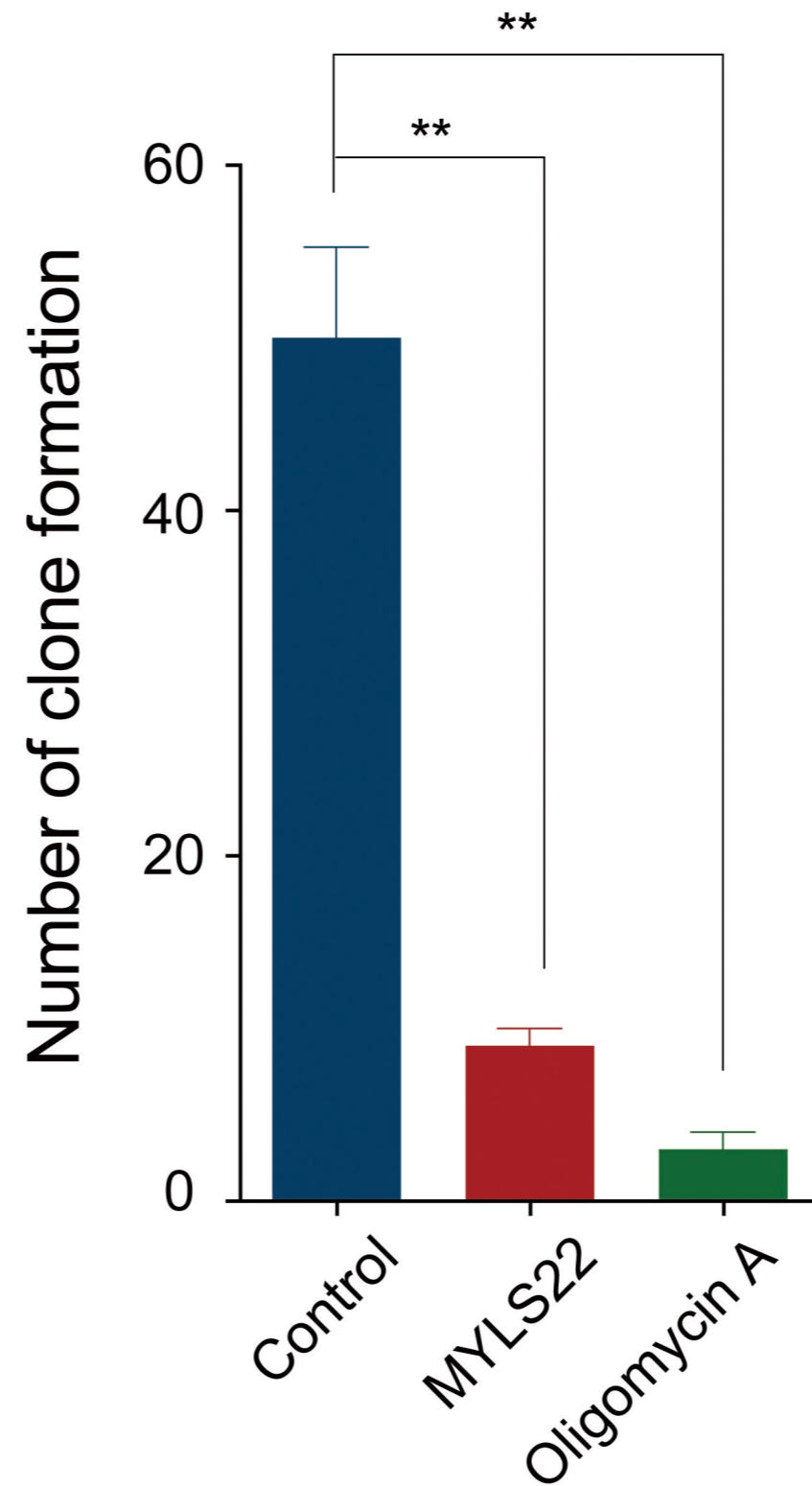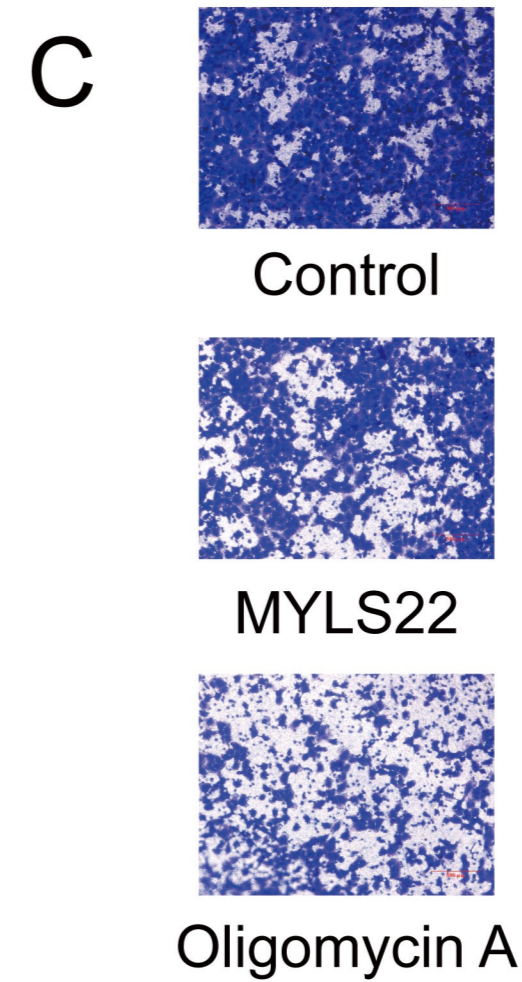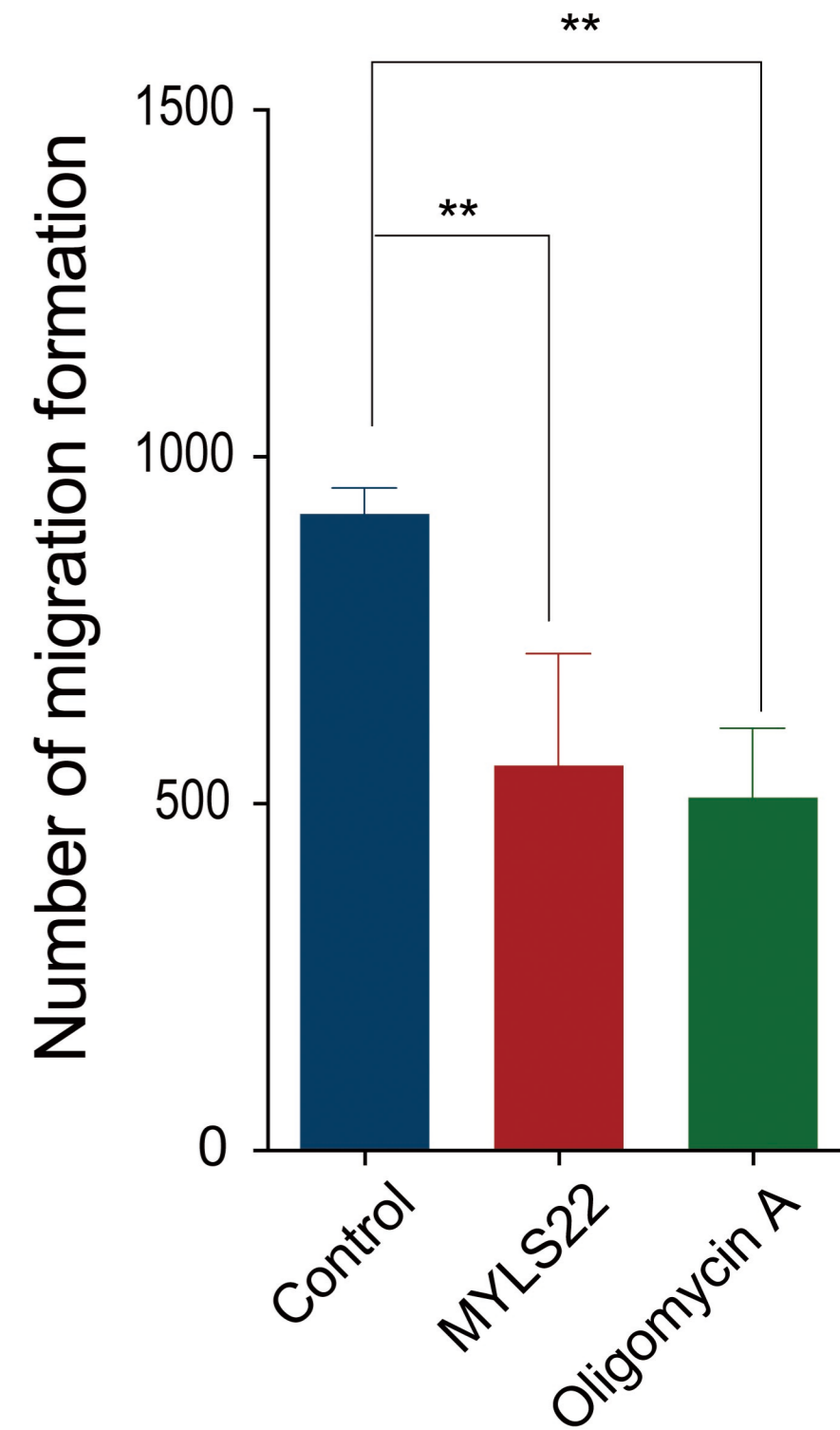

Supplement: Supplemental Information 4 — (A) Cell proliferation was measured by CCK8 assay in Control, MYSL22, and Oligomycin A NCI-H1299 cells. (B) Colonies were counted in Control, MYSL22, and Oligomycin A NCI-H1299, statistical results are present on right. (C) Representative images of transwell assays (migration) in Control, MYSL22, and Oligomycin A NCI-H1299 cells. Statistical results are present on right. [file peerj-10-14543-s004.pdf]
